# Chronic Expanding Hematoma in the Upper Extremity: A Case Report on Diagnostic and Management Challenges

**DOI:** 10.7759/cureus.62330

**Published:** 2024-06-13

**Authors:** Oluwatomisin M Olapoju, Joseph Botros, Bobbie Damani, Frederick Tiesenga

**Affiliations:** 1 Surgery, Richmond Gabriel University, Kingstown, VCT; 2 Surgery, St. George's University School of Medicine, True Blue, GRD; 3 General Surgery, West Suburban Medical Center, Chicago, USA

**Keywords:** swelling, soft-tissue malignancies, anticoagulant therapy, chronic expanding hematoma, hematoma

## Abstract

Hematomas are a common occurrence in clinical practice, often resulting from trauma or underlying bleeding disorders. They can manifest with various symptoms depending on their location and size. While hematomas are typically straightforward to diagnose and manage, specific presentations can pose diagnostic challenges. We present the case of a growing post-traumatic right upper extremity mass in a 67-year-old male on anticoagulant medication, Xarelto (rivaroxaban) 20mg, with no history of bleeding disorders. Differential diagnoses include a benign lipoma or possible soft-tissue malignancy. The mass was surgically excised and sent to pathology, which confirmed it was a hematoma and led to a diagnosis of a chronic expanding hematoma. Ultrasonography, computed tomography, and magnetic resonance imaging are diagnostic modalities that can help distinguish different presentations. Despite the commonality of hematomas in clinical practice, atypical hematomas may also present unique challenges in diagnosis and management due to their varied clinical presentations and locations. This report underlines the importance of understanding hematomas' diverse etiologies, presentations, and imaging characteristics for appropriate treatment and diagnosis.

## Introduction

A hematoma is a collection of blood that forms in a tissue, organ, or body space [1]. It often occurs due to trauma, underlying bleeding disorder, or after surgery. Depending on the surrounding tissue, it can occur anywhere in the body and may become a more significant concern. According to Snyder et al. 2009, hematomas adjacent to the elbow should be followed closely due to concerns of immobility [2]. Most hematomas are typically small and resolve with time, but some may require surgical intervention, as in this patient's case. Risk factors for developing a hematoma include genetic conditions like hemophilia or von Willebrand's disease or anticoagulant medications such as warfarin and apixaban. These factors reduce the blood's ability to form clots, making it more difficult to stop bleeding and, thus, more likely to develop a hematoma [3]. Despite the commonality of hematomas in clinical practice, atypical hematomas may present unique challenges in diagnosis and management due to their varied clinical presentations and locations. We present a case of a growing post-traumatic right upper extremity hematoma in a male patient on anticoagulant medication with no history of bleeding disorders. This report highlights the etiology, associated risk factors, and diagnosis of chronic expanding hematomas. It emphasizes differential diagnosis and the need to thoroughly evaluate and manage atypical cases.

## Case presentation

**Figure 1:**
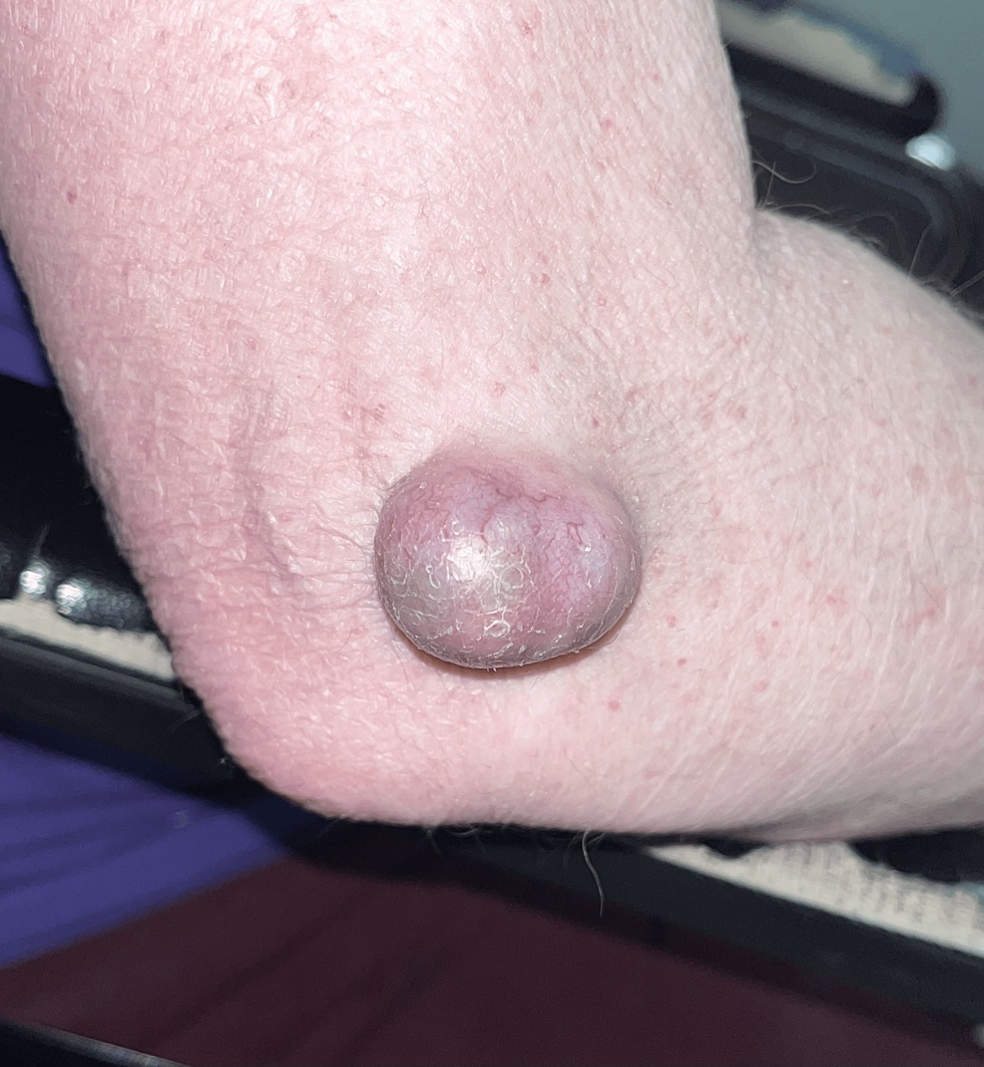
Right elbow mass

The patient was a 68-year-old male with a medical history significant for a stroke, for which the patient had been on Xarelto 20mg for about eight years. He presented to our outpatient clinic with complaints of a mass on the posterior aspect of his right elbow that appeared over a year ago when he bumped his arm in the nursing home where he lives. The patient denied any similar history and noted that the mass had gradually increased. He expressed that the mass had become bothersome as he kept bumping his hand into the wall, which would cause pain. Examination showed a firm, immobile mass with light streaks of blood vessels on the surface and no drainage (Figure 1). It measured approximately 3.6 cm x 2.1 cm x 2.0 cm.

**Figure 2:**
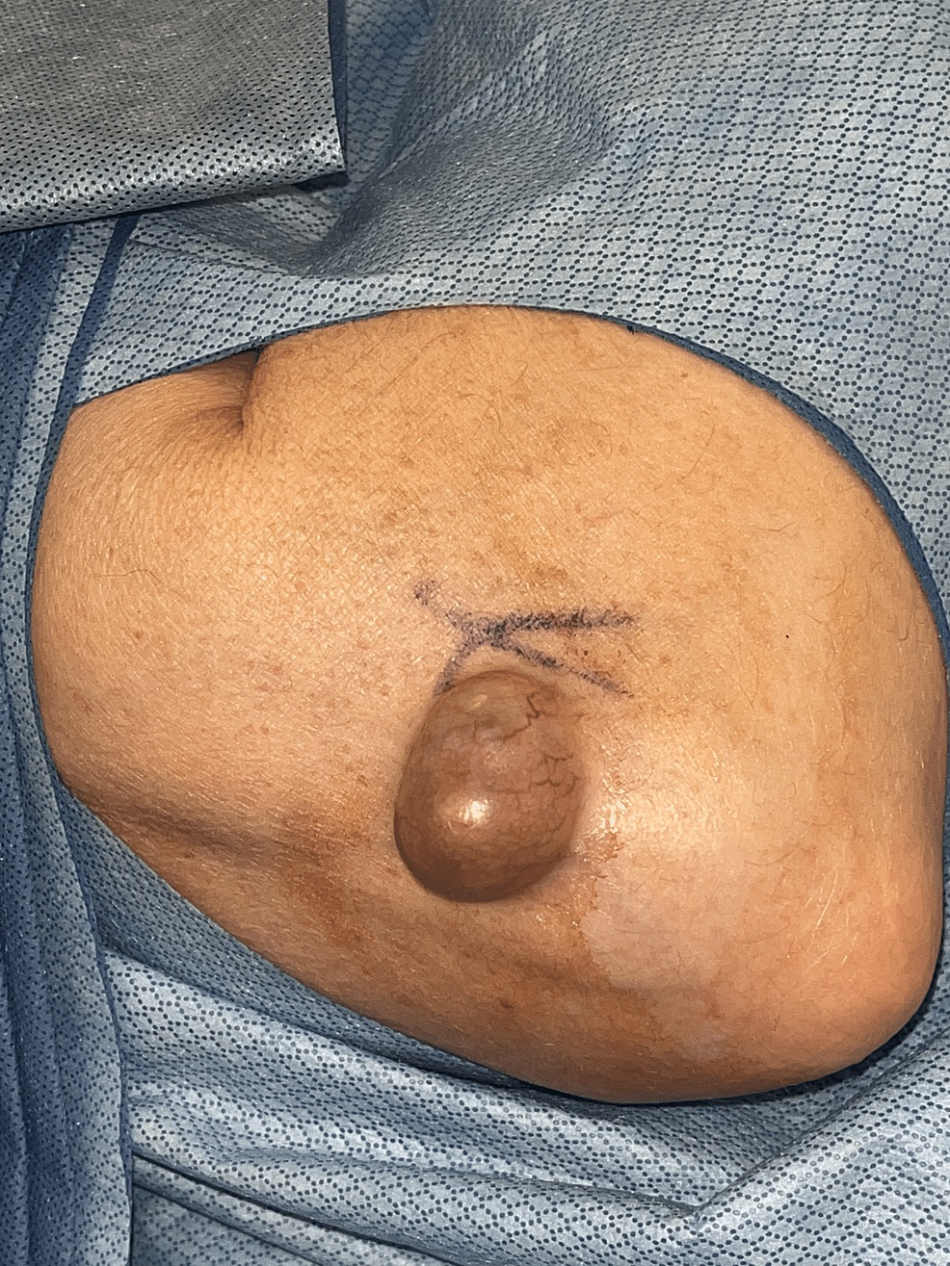
Marked surgical site

**Figure 3:**
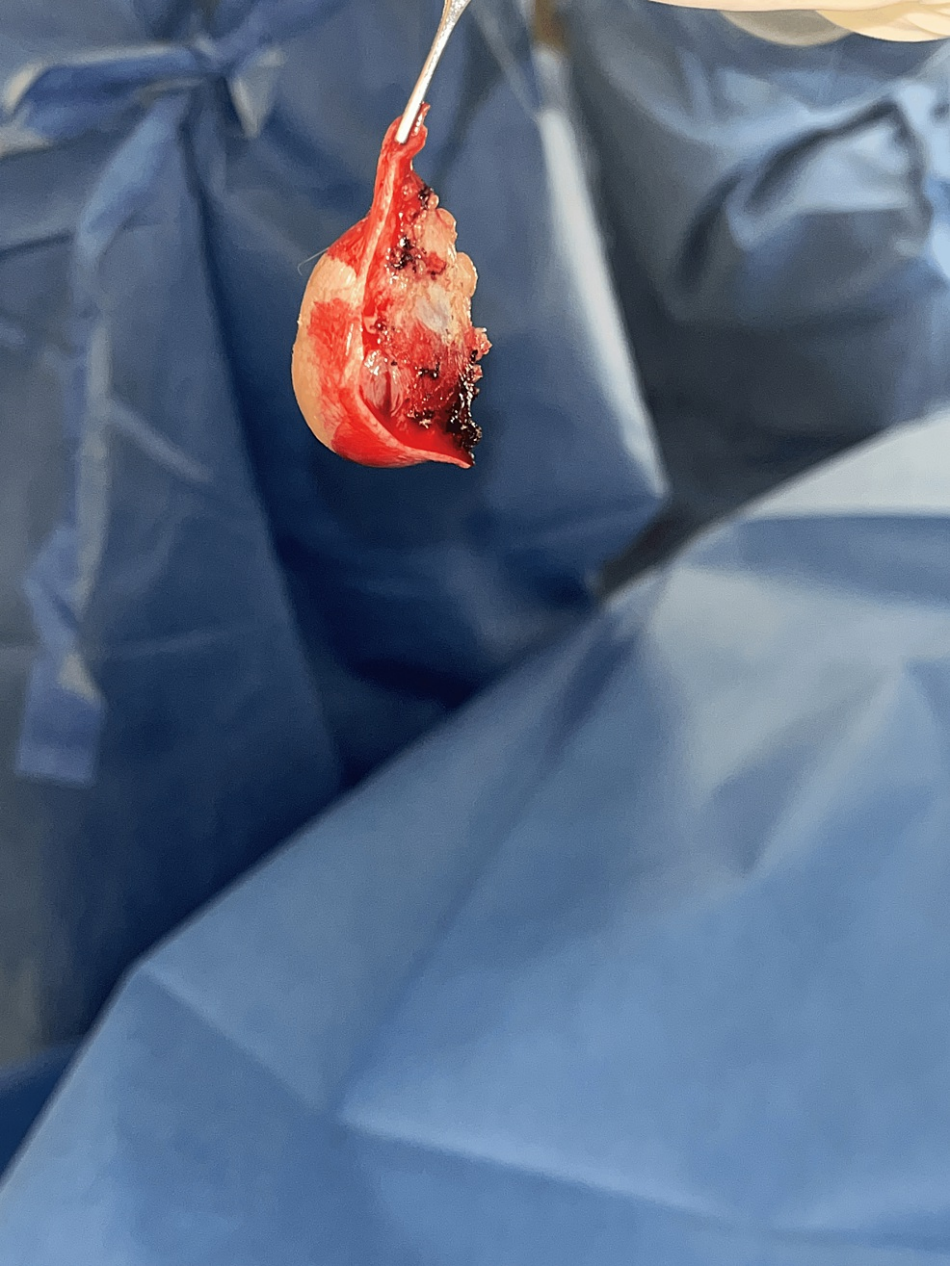
Excised mass

**Figure 4:**
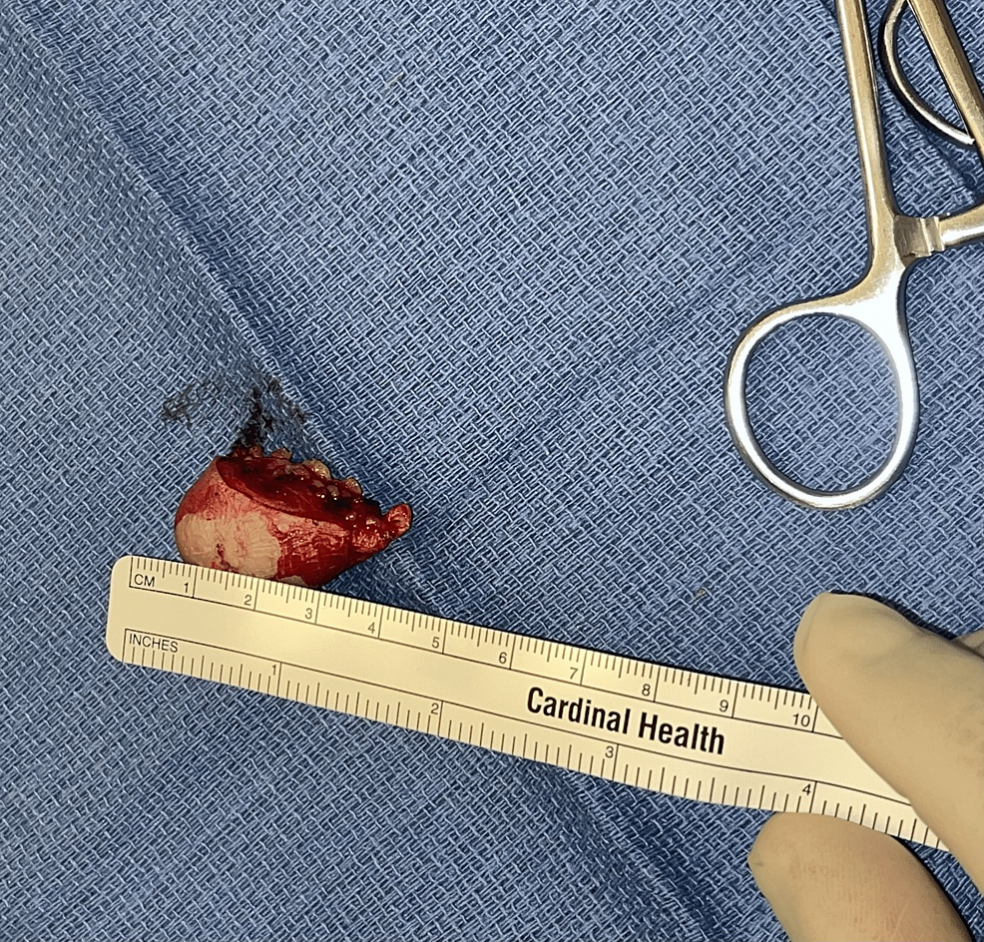
Gross appearance before histopathology workup

After history and physical examination, no medical imaging was conducted due to the patient's decision. The patient was scheduled for an excision of a right elbow subcutaneous mass, and Xarelto was stopped three days before the surgery. The patient was assessed and cleared. At surgery (Figure 2), the mass was excised (Figure 3) without complications and sent to pathology (Figure 4).

Histopathologic results showed that the interior of the mass was utterly hemorrhagic, consistent with an organized hematoma.

## Discussion

A hematoma is a collection of blood that forms outside a blood vessel in the body [1]. This appears as skin discoloration, swelling, or ecchymosis. Numerous potential causes of hematomas exist, such as vitamin deficiencies, clotting factor deficiencies like hemophilia, chronic liver disease, anticoagulant usage, malignancy, and trauma. Research has indicated that long-term warfarin use [4] and apixaban [5] increase the likelihood of spinal epidural hematomas. While no direct evidence links rivaroxaban (as in this patient) and soft-tissue hematomas, individuals on anticoagulants are at risk for bleeding and, therefore, developing hematomas.

Although most hematomas undergo spontaneous resolution without sequelae, a subset demonstrates atypical behavior by persisting and evolving into slowly enlarging masses resembling soft-tissue malignancies [6]. These slowly enlarging masses, atypical hematomas are known as chronic expanding hematomas. However, in the case of our patient, pathology results revealed that the mass was not malignant.

Reid et al. (1980, as cited in the paper by Ito et al., 2014) defined a chronic expanding hematoma as "a hematoma, which more than one month after initial hemorrhage, is characterized by its persistence and increasing size" [6]. The precise etiology of chronic expanding hematoma (CEH) remains unclear and may occur spontaneously or as a result of prior trauma or surgical intervention. One possible explanation suggests that initial trauma disrupts the integrity of the skin and subcutaneous fat, creating a cavity that fills with blood and becomes encapsulated by dense fibrous tissue. Chronic inflammation within this capsule, fueled by the breakdown products of leukocytes, erythrocytes, and platelets, is hypothesized to perpetuate injury and bleeding from nearby capillaries. Several kinds of literature show CEHs occur in other body parts such as the chest, retroperitoneal space, buttocks, thigh, knee, and even the soles of the feet [6-9].

Accurate diagnosis of a chronic expanding hematoma begins with a thorough history and physical examination of a patient, followed by imaging such as ultrasound, computed tomography (CT) scan, or magnetic resonance imaging (MRI). MRI has gained recognition as a valuable tool for diagnosing hematomas due to its ability to differentiate fluid and soft-tissue signatures. CEHs are typically characterized on MRI by the presence of a pseudocapsule [9]. This finding corresponds histologically to a capsule composed of fibrous tissue containing hemosiderin deposits and macrophages laden with iron.

A biopsy could also be done to identify the cells and rule out a neoplasm. Distinguishing CEH from soft-tissue sarcomas (STS) can be challenging due to potential misdiagnosis with ultrasound and MRI. Prior literature documented cases of misdiagnosis for both lesions [10]. The limitations of ultrasound and the inherent imaging similarities between CEH and STS on MRI contribute to these diagnostic errors. While advanced imaging can be helpful, even MRI and positron emission tomography (PET) scans may struggle to distinguish chronic hematomas from STS in some cases definitively. However, promising advancements are emerging with diffusion-weighted imaging (DWI) on MRI [10]. This technique potentially leverages variations in apparent diffusion coefficient (ADC) to improve diagnostic accuracy.

The first-line treatment for CEHs is complete surgical removal, as was done in this patient. Depending on the site of the hematoma, it could be challenging to remove entirely due to fibrous adhesions if located in the chest [9]. In the case of our patient, there were no adhesions, and the excision was smooth. Most studies showed no recurrence after one to two years. Only one prior study reported a recurrence rate of 22% (two out of nine patients) following marginal excision of chronic expanding hematomas based on our search on PubMed and Google Scholar [11]. This study has some limitations, including the fact that we did not perform any imaging due to the patient's socioeconomic status. However, the biopsy confirmed the diagnosis.

## Conclusions

In conclusion, the case presented highlights the diagnostic and management challenges associated with chronic expanding hematomas, particularly in patients on anticoagulant therapy. The importance of considering atypical hematomas in the differential diagnosis of soft-tissue masses cannot be overstated. This case emphasizes the significance of thorough clinical evaluation, appropriate imaging studies, and histopathologic analysis in establishing an accurate diagnosis and guiding the management of such challenging cases. Further research and clinical experience in the field of hematomas, especially chronic expanding hematomas, are warranted to better understand their etiology, behavior, and optimal management strategies. This will contribute to improved outcomes for patients presenting with similar clinical scenarios.

## References

[1] (2011). National Cancer Institute: Hematoma. https://www.cancer.gov/publications/dictionaries/cancer-terms/def/hematoma.

[2] Snyder MJ, Hicks BM, Thieken M (2009). Traumatic distal humeral hematomas: a report of 2 cases. Am J Orthop (Belle Mead NJ).

[3] Fior D, Di Provvido S, Leni D (2023). Spontaneous soft tissue hematomas in patients with coagulation impairment: safety and efficacy of transarterial embolization. Tomography.

[4] Li X, Zeng Z, Yang Y (2022). Warfarin-related epidural hematoma: a case report. J Int Med Res.

[5] Mezzacappa FM, Surdell D, Thorell W (2020). Spontaneous spinal epidural hematoma associated with apixaban therapy: a report of two cases. Cureus.

[6] Ito T, Nakahara T, Takeuchi S, Uchi H, Takahara M, Moroi Y, Furue M (2014). Four cases of successfully treated chronic expanding soft tissue hematoma. Ann Dermatol.

[7] Sakuma T, Takayashiki N, Iguchi K, Kagohashi K, Satoh H, Nakazawa K, Hizawa N (2018). Chronic expanding hematoma in the chest: a case report. Exp Ther Med.

[8] Syuto T, Hatori M, Masashi N, Sekine Y, Suzuki K (2013). Chronic expanding hematoma in the retroperitoneal space: a case report. BMC Urol.

[9] Sakamoto A, Okamoto T, Matsuda S (2017). Chronic expanding hematoma in the extremities: a clinical problem of adhesion to the surrounding tissues. Biomed Res Int.

[10] Wells ME, Qiao J, Decker KE, Parnes N, Rajani R, Eckhoff M (2023). A masquerading hematoma resulting in the delayed diagnosis of a soft tissue sarcoma: a case report. Cureus.

[11] Okada K, Sugiyama T, Kato H, Tani T (2001). Chronic expanding hematoma mimicking soft tissue neoplasm. J Clin Oncol.

